# Biochemical, Physicochemical and Sensory Properties of Yoghurts Made from Mixing Milks of Different Mammalian Species

**DOI:** 10.3390/foods9111722

**Published:** 2020-11-23

**Authors:** Oumayma Boukria, El Mestafa El Hadrami, Aysha Sameen, Amna Sahar, Sipper Khan, Jasur Safarov, Shakhnoza Sultanova, Françoise Leriche, Abderrahmane Aït-Kaddour

**Affiliations:** 1 Applied Organic Chemistry Laboratory, Sciences and Techniques Faculty, Sidi Mohamed Ben Abedallah University, BP 2202 Route d’Immouzer, Fez 30050, Morocco; boukriaoumayma@hotmail.com (O.B.); elmestafa.elhadrami@usmba.ac.ma (E.M.E.H.); 2 National Institute of Food Science and Technology, University of Agriculture, Faisalabad, Punjab 38000, Pakistan; ayshasameen@uaf.edu.pk; 3 Department of Food Engineering/National Institute of Food Science and Technology, University of Agriculture, Faisalabad, Punjab 38000, Pakistan; 4 School of Food and Agricultural Sciences, University of Management and Technology, Lahore 54770, Pakistan; sipperkhan@gmail.com; 5 Department of Food Engineering, Faculty of Mechanical Building, Tashkent State Technical University Named after Islam Karimov, University Str. 2, Tashkent 100095, Uzbekistan; jasursafarov@yahoo.com (J.S.); sh.sultanova@yahoo.com (S.S.); 6 Université Clermont Auvergne, INRA, VetAgro Sup, UMRF, F-63370 Lempdes, France; francoise.leriche@vetagro-sup.fr

**Keywords:** milk, mixture, yoghurt, nutrition, rheology, sensory, camel milk, cow milk, ewe milk, goat milk

## Abstract

Among developed countries, bovine milk production makes a major contribution towards the economy. Elevating consumer demand for functional foods has triggered a niche for non-bovine milk-based products. Mixing milks from different species can be a strategy to increase the consumption of non-bovine milk and enable consumers and dairy companies to benefit from their nutritional and technological advantages. Thus, this review aimed to gather the most important research on yoghurts derived from processing mixtures of milks of different species. We discuss the impact of milk mixtures (i.e., species and milk ratio) on nutritional, physicochemical, sensory, rheological and microbiological properties of yoghurts. More specifically, this paper only highlights studies that have provided a clear comparison between yoghurts processed from a mixture of two milk species and yoghurts processed from a single species of milk. Finally, certain limitations and future trends are discussed, and some recommendations are suggested for future research.

## 1. Introduction

Over the past decade, escalating consumer interest has been reported towards functional foods. This market change is associated not only with high nutritional values but also with their health promoting constituents that consequently reduce the risk of several diseases [1]. Milk and dairy products are one of the potential categories of resources for providing functional food products, due to their content in a variety of essential components. Every mammal species has a unique milk composition in terms of major and minor constituents such as proteins, polyunsaturated fatty acids (FAs), vitamins, and minerals [2,3,4]. The simplicity of incorporating probiotic bacteria (LAB: lactic acid bacteria) during manufacturing enables the production of bioactive components in milk including acid γ-aminobutyric (GABA) [5]. Major contributions in the dairy economy come from cow milk (CM). This milk represents ca. 83% of the total world milk production [6]. Nonetheless, milking animals are not limited to cows in many parts of the world and, thus, buffalo (13% of world production), goat (2.2%), ewe (1.3%), and camel (0.3%) milks are also available in significant quantities [7,8]. 

Camel milk (CaM) presents a high nutritional quality, e.g., it has 3 times more vitamin C, minerals (e.g., K^+^), and essential and polyunsaturated FAs than cow milk (CM) [9,10]. It also contains many antimicrobial agents (lysozyme, lacto-peroxidase, lactoferrin, immunoglobulins and bacteriocins) that participate to its high bacteriostatic property [1]. Goat milk (GM) is generally reported to have a higher fat and protein content than CM, with lower α s1 –CN resulting in a fine and softer curd, thereby lowering its allergenic potential. Similarly, GM is also recognized to have smaller fat globules and to contain higher concentration of medium chain FAs as compared to CM [11,12,13,14,15]. It also contains higher levels of vitamin A, thiamine and niacin [16]. Studies have also shown that it has higher quantities of β-casein, lower quantities of αs-casein, and nearly identical quantities of κ-caseins [17] and it exhibits higher content of α-tocopherol/vitamin E than CM [18]. 

GM is known to be advantageous in terms of intolerance in allergic children compared to CM. The anti-allergic property of GM is principally associated with the structure and quantity of two major allergens: α-lactalbumin and β-lactoglobulin [15,16,17]. Moreover, GM presents higher Ca^2+^, vitamins (e.g., A and B complex), and oligosaccharide contents, with higher amounts of short FAs and conjugated linoleic acid providing better digestibility. It can also be seen as a natural source of oligosaccharides [18,19,20], which implies that it provided many health benefits compared to CM [21]. However, GM exhibits higher saturated FAs and lower polyunsaturated FAs contents, attributed to the development of heart disease.

Regarding ewe milk (EM), it is described as an excellent source of nutrients thanks to its high amount of essential amino acids (AAs) [22]. Differences in composition between CM and EM mainly related to the high percentage of proteins and fats reported in EM, explain the various technological and sensorial properties of yoghurts [23]. It is rich in small fat globules. Lipolysis in EM is faster as compared to CM, which contributes to the development of an important and typical flavor [15] owing to a higher short-chain FAs contents namely caproic (C6:0), caprylic (C8:0), and capric (C10:0) acids [24]. As compared to CM, buffalo milk (BM) was found to be richer in lactose, fat, protein (especially caseins), inorganic phosphate, vitamins and minerals (e.g., calcium, magnesium) [25,26] (Table 1). 

Despite the nutritional benefits of the aforementioned non-bovine milks, their consumption and utilization in processing of dairy products is scarce in some countries (such as in Brazil) [49]. Globally, non-bovine milk contributes to 133 million tons per year, resulting in 17% of the total milk production [50]. Therefore, the countries not involved in the commercial production of non-bovine milk product are unable to monetize from this opportunity [51]. However, for some of the derived products, the flavour of these milks is distinctive and stronger than CM, which constrains its acceptability among many consumers [52,53]. Although mixtures from different species contribute to the overall nutritional profile, many countries, including European countries, do not allow this practice legally [54]. However, some economic impacts of milk mixtures make this strategy feasible and interesting from the farmer’s perspective [55]. In this context, the production of dairy products using mixtures of milk species (e.g., GM with CM) could be an interesting and feasible strategy to promote and expand the dairy industry in many regions, strengthening the non-CM production chain. Moreover, optimized mixing proportions can also improve the quality of fermented dairy products and develop new ones with specific nutritional (biochemical), physicochemical, sensory and rheological properties. As reported by Aryana and Olson [56], yoghurt is one of the most popular food products in the world with an increased annual consumption worldwide, owing to the nutritional profile of this dairy product [56]. An increase in yogurt consumption from 0.1 kg in 1970 to 1.2 kg in 1977 was observed in the USA [57], while the production of yoghurt increased from £ 982.6 million in 1990 to about £ 4,742.1 million in 2015 [56]. Today, many forms of yoghurt can be found including plain yoghurt, fruit flavoured yoghurt (including fruit-on-the-bottom and blended forms), whipped yoghurt, granola-topped yoghurt, drinkable yoghurt, frozen yoghurt, and Greek yoghurt with varying fat contents (regular, low fat, and non-fat) [56]. The shelf life and quality of yoghurt are determined by changes in the physical, chemical and microbiological characteristics, which modify the organoleptic properties and likewise decrease product quality and consumer acceptability [58,59].

Previously, no study has been found reviewing the quality of yoghurts prepared from different mammalian milk proportions. Therefore, the aim of the present review is to gather the different studies performed on mixing milk from different species and discuss the effect on chemical, texture and sensory properties of the resulting yoghurts (Table 2). Actual examples of processed yoghurts will be compared and discussed throughout the review as well as their quality features. More specifically, this review focuses on studies that provide a clear comparison between yoghurts produced from one milk species and yoghurts produced from a mixture of different milk species. 

## 2. Approximate Composition of Yoghurt

As far as we know, most of the studies previously reported in the literature were performed after considering mixtures of EM with other mammalian milk species, like GM, CM and CaM (Table 3). The previous literature examined the similar effects of milk and related products from single species, e.g., cow or camel milk only.

A research study done in 2004 [65] investigated the effect of mixing EM and GM on yoghurt quality after processing it for a day. In this study, mixing proportions of 1GM:1EM (*v*/*v*-volume/volume) and 2.3GM:1EM, (*v*/*v*) were considered. Insignificant differences were recorded between mixed targeted proportions, pure EM and GM products in terms of lactose, total solids, fat, proteins, ash, and minerals including calcium, magnesium, and sodium. The insignificant differences were reportedly linked to the usage of standardized milk with stable protein content before the blending process. Moreover, the mixed types exhibited higher textural properties owing to the high protein and calcium content present in the milk mixtures. In contrast, Bano et al. [67] and Güler and Gürsoy-Balci [63], who studied the same milk blends, reported contradictory results. Bano et al. [67] highlighted that adding EM into GM increased fat, protein, lactose, ash and total solids for different yoghurt formulations (3GM:1EM, 1GM:1EM, and 1GM:3EM, *v*/*v*) regardless of the storage time (28 days); while Güler and Gürsoy-Balci [63] reported an increase in total solids, fat, protein and ash for yoghurt containing a mixture of both sources added equally. 

As previously reported, Vianna et al. [66] noted equivalent conclusions when CM was added to EM. They reported an increase in total solids, protein, lipid and ash for the following yoghurt formulations, 3CM:1EM (*v*/*v*), 1CM:1EM (*v*/*v*), and 1CM:3EM (*v*/*v*). 

In a similar approach, Ibrahem and Zubeir [64] substituted CM with CaM and reported differences in the approximate composition of different yoghurt formulations, pure CaM, 1.5CaM:1EM, and 1CaM:1.5EM. They highlighted that total solid content decreased when decreasing the percentage of EM in the mixtures. Concerning protein content, they reported variations between yoghurts during storage. Concerning ash, no differences were highlighted [71]. Conclusively, all the above-mentioned studies assigned variations observed in yoghurt containing milk mixture to EM proximate composition [72,73]. This was essentially due to its higher content of total solids [15], proteins [23], minerals [72], and lipids [15,74], when compared to CM, CaM and GM [71].

The detailed composition of yoghurt was provided only by Güler and Gürsoy-Balcı [63]. These authors reported that the type of milk (i.e., GM and EM) significantly influenced yoghurt composition. They found that the acetaldehyde content of 1GM:1EM (*v*/*v*) yoghurts were at intermediate level, between those of EM and GM yoghurts, while EM yoghurt presented the lowest level. The difference observed may be accounted for by the amino acid composition of EM [75]. Concerning diacetyl content, 1GM:1EM (*v*/*v*) yoghurts had the highest level when compared with the other yoghurts. Moreover, it was observed that diacetyl content steadily decreased in GM yoghurt during storage, while the other yoghurt formulations exhibited an inverse trend. Concerning acetoin, the mean level was higher in 1GM:1EM (*v*/*v*) yoghurt, followed by GM and EM yoghurts. GM (5.82 g/100 g ± 2.14) presented the highest ethanol content followed by 1GM:1EM (4.40 g/100 g ± 1.64) and then EM (2.83 g/100 g ± 1.08). The difference was assigned principally to the type of milk since ethanol was highest in unfermented GM when compared with the other milk formulations. The changes in ethanol were similar to the variations of ethanoic acid levels in the experimental yoghurts during storage [76]. 

Concerning the total amount of free fatty acids (FFAs), no significant difference was observed in EM, GM and 1GM:1EM yoghurts [63,77]. Nonetheless, when the detailed analyses of FFAs were performed, they highlighted some differences. Short chain FFAs (i.e., butanoic, hexanoic and octanoic acids) in all yoghurts were significantly affected by the type of milk. The total amount of short-chain FFAs (C4–C8) was significantly higher in EM yoghurt than in the other types of yoghurt. This was assigned to differences in characteristic FFAs in milk fat between types of milk, since short chain FFAs, especially C4, was markedly high in EM in comparison with GM [77,78]. With respect to medium-chain FFAs, the levels of decanoic and dodecanoic acids were lower in yoghurts containing equal proportion of GM and EM. Tetra-decanoic acid resembles butanoic and hexanoic acids, which was significantly higher in EM yoghurt than in others. This could be attributed to high tetra-decanoic acid content in EM in comparison with GM, which was observed by others [77,78]. Concerning long-chain FFAs, the type of milk did not affect their level in the yoghurts.

The effect of GM and CM mixtures on yoghurt’s approximate composition was investigated previously only by Serhan et al. [69]. In this study, they combined the approach of Bano et al. [67] and Vianna et al. [66] in terms of milk formulations. Their research was centred on the manufacturing of concentrated yoghurt (Labneh). When comparing Labneh prepared with CM and GM, the latter contained higher moisture, ash and fat contents and lower total solids, protein and lactose contents. Regarding mixtures, they highlighted that Labneh containing 20%, 30%, 40% and 50% (mass fraction) of GM presented intermediate moisture, protein, fat and ash concentrations. Considerable difference in fat and protein content was observed in both CM and GM Labneh, owing to the compositional properties of the milk employed. CM is reportedly rich in both fat and protein constituents [79,80]. Nonetheless, these conclusions did not corroborate with the difference in approximate composition exhibited in the review of Clark and Mora-García [81].

## 3. Acidity and pH of Yoghurt

The control of pH and acidity are undoubtedly important parameters in yoghurt processing due to their functional contribution in curd coagulation, ripening, and shelf life. In yoghurt the decrease of pH by the fermentation of lactose to lactic acid by LAB reduces the electrostatic repulsion between casein micelles and alters the distribution of Ca between the micelle and serum phases [82,83,84]. Therefore, different milk mixtures are needed to optimize the obtaining of effective acidity and pH. These mixtures will also assist in the prevention of syneresis in yogurt. 

One of the pioneer investigations that tackled the effect of mixing milk from different species was conducted by Kaminarides and Anifantakis [65]. They compared pure GM yoghurts prepared from milk coming from two breeds, including an imported Alpine breed and local Thiva breed (Athens, Greece). The yoghurts included pure EM yoghurts obtained from mixtures of the two species (1GM:1EM and 2.3GM:1EM, *v*/*v*). Initially, they reported that the comparison of milk mixtures (1GM:1EM and 2.3GM:1EM) and pure milks (only GM and EM) exhibited no differences in pH. This was also confirmed when the mixtures were compared for both acidity and pH. As expected, the same conclusions were reported for yoghurts obtained at 5 °C after one day of storage. These results were in accordance with those of Bano et al. [67] who compared yoghurts produced with pure GM, pure EM, and mixtures of the two (3GM:1EM, 1GM:1EM and 1GM:3EM, *v*/*v*). They also observed that the addition of GM into EM does not affect the acidity and pH of yoghurts. Nonetheless, contrary to Kaminarides and Anifantakis [65], their conclusions were reported after considering pure buffalo milk yoghurt as being the control, and not the pure EM and the pure GM yoghurts. In the same year, Güler and Gürsoy-Balci [63], after studying equivalent milk mixtures (1GM:1EM, *v*/*v*) combined with different starter LAB (i.e., CH-1 and YF-3331), reported significant differences between yoghurts. During storage (1 to 21 days at 4 °C), the lowest pH and the highest titratable acidity values were obtained from EM yoghurt with culture CH-1. On the other hand, the highest pH and lowest titratable acidity values were found in 1EM:1GM (*v*/*v*) yoghurt with culture YF-3331 at day one. They pointed out that the starter culture’s activity and growth rate varied in accordance with the type of milk [85]. They also reported that post acidification was highly affected by interaction between type of milk and time of storage. The pH and titratable acidity values of yoghurts were significantly influenced by the culture used. This result matched with the findings of others [70,77,78]. Nonetheless, these authors noted that there were no significant differences in incubation time among the types of milk suggesting that the final adequate pH of the yoghurt was achieved in the same period by all the formulations. 

When replacing EM by CM in the mixture (i.e., 3GM:1CM, 1GM:1CM, 1GM:3CM, –% mass fraction–) Vargas et al. [60] observed an effect on the pH of yoghurt samples during storage. Indeed, after one day of cold storage (4 °C), the yoghurts containing pure GM and a high proportion of GM (i.e., 3GM:1CM) reached the lowest pH value (pH = 4.1), which remained constant during storage (28 days). Moreover, they also noted that 1GM:1CM and 1GM:3CM samples (i.e., containing 50% or less of GM) showed the slowest pH decrease reaching a practically constant value (ranging between 3.9 and 4.1) after 14 days. Kücükcetin et al. [61] reported equivalent results for stirred yoghurts (1GM:1CM) stored at 4 °C for 15 days. Recently Serhan et al. [69] also confirmed those observations after studying different types of Labneh prepared with the following concentrations: pure GM, pure CM, 1GM:1CM, 1GM:1.5CM, 1GM:3.5CM, 1GM:4CM, and 1GM:9CM (*v*/*v*), all stored at 4 °C after manufacturing. They concluded that incorporating GM into CM seems to decrease pH and increase the rate of acidification [86,87]. These observations were assigned to the enhancement of microbial growth, acidity progress and peptidase activity of *L. delbrueckii* subsp. bulgaricus in GM [88]. However, the acidification rate of LAB varied with the type of milk; some yoghurt starters are more active in GM while others were reportedly more active in CM, regardless of the starter type (Table 4).

A modification of the yoghurt pH was also noted by Ibrahem and Zubeir [64,71], who investigated for the first time the effect of mixing CaM and EM on yoghurt during cold storage (29 days at 4 °C). The mixtures studied included pure CaM, 1.5CaM:1EM (*v*/*v*), and 1CaM:1.5EM (*v*/*v*) with same starter cultures used in both pieces of research. The starter culture included s1: (*Streptococcus thermophilus* and *Lactobacillus delbruckii* subsp. *Bulgaricus*) (YC-X11 Thermophilic Yoghurt Culture – Yo-Flex CHR HANSEN) used for textural properties and s2: (CH-1 Thermophilic Yoghurt Culture –Yo-Flex CHR HANSEN) for imparting acidity and flavour characteristics. The evaluation of titratable acidity demonstrated that yoghurt samples containing a mixture of CaM and EM presented an intermediate acidity value compared to yoghurts produced only from CaM (exhibiting the lowest acidity) and EM (demonstrating the highest acidity). Their results evinced that increasing the content of EM increased the development of acidity among yoghurt samples, with the highest acidity value obtained in the 1CaM:1.5EM sample. The highest acidity of EM yoghurts was related to higher buffering capacity due to the increase in protein content present in the milk used [89]. Moreover, CaM is reported to be slow in acidification due to the presence of inhibitory substances, resulting in reduction and prevention of microbial growth [90]. However, it has been recently reported that this slow acidification is due to limited proteolysis of CaM proteins by the starter cultures rather than microbial inhibitory substances present in the milk product [91]. 

*Streptococcus thermophilus* and *Lactobacillus delbrueckii* subsp. *bulgaricus* are LAB used for yoghurt production [75,88]. Different starters (e.g., commercial CH-1 and YF-3331) are used for set types of yoghurt production but these starters contain strains at different ratios [92] (Chr. Hansen’s Technical Bulletin, 2004). In this context, limited published data on LAB’s activity in milk from different species is available. For example, Uysal et al. [62] used mixtures of caprine and bovine milks (1GM:2.33CM and 1GM:1CM) to manufacture bio-yoghurt fortified or not with milk concentrate. Bio-yoghurts are reported to contain live probiotic bacterial cultures including *Lactobacillus acidophilus* and *Bifidobacterium bifidum*. After 14 days storage, no significant difference could be observed between bacterial (*Lactobacillus acidophilus, S. thermophilus, Bifidobacterium bifidum*), yeast and mould counts according to the proportion of different milks. Vianna et al. [66] described similar results for the enumeration of the inoculated bacterial cultures in five probiotic yoghurt samples prepared using solely CM, EM or their mixture at different proportions. On the first day of storage, bacterial count (*Lb delbrueckii* spp. *bulgaricus, Streptococcus thermophilus and Lb acidophilus*) for CM was 10.0, 13.8 and 8.7 log (colony forming unit / gram) (cfu/g), while for 3CM:1EM bacterial count was observed to be 9.7, 13.7 and 9.3 log cfu/g, and for 1CM:1EM count was 9.6, 13.6 and 9.3 log cfu/g. Similarly, for 1CM:3EM bacterial count of 10.2, 13.5 and 8.9 log cfu/g and for EM, 9.2, 13.8 and 8.8 log cfu/g, respectively, was reported. These results highlighted that increasing EM in the mixture decreased the development of the aforementioned bacteria. Contrary to the results reported on storage, a nonlinear relationship was observed between the content of the milk species in the mixture used for yoghurt processing after 28 days of storage. Indeed, (Table 5) summarizes the bacterial population exhibited in different yoghurt samples.

## 4. Sensory Properties of Yoghurt

As aforementioned in this review paper, mixing different milk species significantly influences the physicochemical, biochemical, microbiological, and structural features of yoghurt. These effects can modify either positively or negatively in terms of flavour, aroma, colour, texture, viscosity and water holding capacity (WHC). In this context, several research studies have been published with the objective of estimating the possible alteration in the sensory properties of yoghurt prepared by using milk mixtures from different species [60,68,69,93,94,95,96]. 

Flavour and texture are the most pronounced factors that influence quality and acceptability of yoghurt and related fermented milk and milk products. Many parameters affect the flavour and texture of yoghurt including type of starter culture, incubation temperature, processing conditions (e.g., heat treatment, homogenization), compositional properties of the milk and probiotics addition [5,81,97,98,99,100]. Therefore, detailed control of these characteristics is needed for acceptability of yoghurt. It is recognized that yoghurt should have a fine and smooth texture along with a firm body to hold its shape when it is spooned [101]. The flavour should be clean, distinct, and acidic [56]. The final yoghurt flavour is generally assigned to several compounds, e.g., non-volatile acids (lactic or pyruvic), volatile acids (butyric to acetic) carbonyl compounds (acetaldehyde to diacetyl) and miscellaneous compounds (amino acids in products formed by thermal degradation) [94]. Lactic acid, acetaldehyde (considered as a key compound) and diacetyl concentrations, along with their relative proportions, are considered essential for the final aroma of the product [75]. 

As early as 2004, Kaminarides and Anifantakis [65] reported that yoghurts prepared from mixtures of GM and EM (1GM:1EM and 1.5GM:1EM, *v*/*v*) presented good quality. Indeed, adding EM to GM improved yoghurt’s texture, serum separation, overall acceptability and firmness. These results were equivalent to those of Bano et al. [67] and Kaminarides and Anifantakis [65]. They noted that the addition of EM (up to 25%) to GM improved the flavour, colour, texture and overall acceptability scores of the yoghurt. However, beyond this level, they identified that it negatively affected flavour. The yoghurts were also considered to be too creamy and criticized for their buttery flavour by some panellists. The decrease in flavour score with the increase in EM was attributed to the presence of FFAs. These FFAs generally appear after milk fat lipolysis. FFAs are generally reported as being more abundant in small ruminant milk fat than in CM [102,103] (Table 6).

Furthermore, Kücükcetin et al. [61] evaluated the effect of adding GM to CM and its contribution to sensory characteristics. As noted with EM [65,67], they reported that mixing GM with CM has a strong impact on yoghurt’s sensory properties. Yoghurts with equal proportion of GM and CM presented higher viscosity and water holding capacity when compared to pure GM yoghurt. When compared to CM yoghurts, 1GM:1CM samples had a lower number of grains, grain mean perimeter and roughness, suggesting a better, smoother structure, generally appreciated by the consumer. Uysal et al. [62] also noted that 1GM:1CM yoghurt fortified with skimmed CM powder presented better sensory properties when compared to the other mixtures. Serhan et al. [69] corroborated the previous investigations after a detailed sensory analysis, focusing on 22 sensory parameters. The samples contained several mixtures (pure GM, 1GM:1CM, 1GM:1.5CM, 1GM:3.5CM, 1GM:4CM, 1GM:9CM and pure CM, *v*/*v*) for the production of concentrated yoghurt (i.e., Labneh). They identified that products containing 10% GM (1GM:9CM) presented a higher score for appearance, a significant parameter for consumer acceptability. Nonetheless, the sample produced with 1GM:1.5CM reportedly had higher acceptability among the panellists, owing to a less goat like taste. Goat flavour is generally reported as one of the limiting factors in the consumption of GM yoghurt. As reported by Eknæs et al. [104], the development of goat flavour in cold, stored fresh milk is due to FFAs, especially C6:0–C9:0, and volatile branched-chain C9 and C10 as 4–methyl and 4–ethyl–C8 are major contributors to this taste [102,103]. Milk lipolysis and lipoprotein lipase (LPL) activity are well correlated in GM and are most pronounced after peak lactation [105]. 

When a mix of EM and CM was investigated, Vianna et al. [66] reported that pure EM yoghurt or yoghurt containing a high proportion of EM (1CM:3EM, *v*/*v*) exhibited greater firmness, WHC, apparent viscosity and lower spontaneous syneresis values than samples containing a high proportion of CM (i.e., pure CM and 3CM:1EM, *v*/*v*). The 1CM:1EM samples demonstrated intermediate values in textural parameters and exhibited an ideal consistency; while pure CM yoghurt exhibited the lowest consistency, overall impression, appearance and purchase intention scores. The analysis of yoghurt microstructure by scanning electron microscopy (SEM) did not exhibit apparent differences among 3EM:1CM, 1EM:1CM and 1EM:3CM (*v*/*v*) yoghurt microstructures. Nonetheless, Vianna et al. [66] stated that the higher the EM content, the denser the gel structure reported, with underlying and more interconnected protein clusters. This corroborates well with previous studies on yoghurt microstructure [15,106]. 

The aforementioned studies are difficult to compare due to the variation in processing conditions, i.e., storage temperature, and yoghurt formulation. In this context, Bernacka et al. [70] investigated, in the same study, the effect of mixing GM, EM and CM, combining in this way the approaches previously published. They observed that all the yoghurts produced from GM, EM, CM, mixed CM and GM, CM and EM, and EM and GM had differences in colour, smell, taste and consistency. Respondents selected three samples as the most popular in terms of taste, such as the yoghurt made with equal proportion of CM and GM, and EM and CM as previously mentioned [62,65,66]. However, the addition of GM also generated dissatisfaction among consumers in terms of taste. Yoghurts produced from mixed CM and GM were slightly tastier compared to pure GM products. Goat flavour is sometimes regarded as positive but may be a negative feature in cheese or milk at lipolysis levels much lower than those responsible for the rancid–butyric flavour [105]. Nonetheless, GM yoghurts presented the lightest colour, called snow-white, while the CM yoghurt colour was creamy yellow. Finally, yoghurts made from CM, GM and their mixture (1GM:1CM) had the highest curd stability [62,65,66]. 

As far as we know, Ibrahem and Zubeir [64], were the only researchers that tackled the effect of incorporating CaM into the milk mixtures to manufacture yoghurt. During their study, the potentiality of mixing EM and CaM was explored in order to improve the processing properties of CaM yoghurt due to its restricted acidification and coagulation properties. The yoghurts were produced by using two different starter cultures (s1: YC-X11 Thermophilic Yoghurt Culture and s2: CH-1 Thermophilic Yoghurt Culture). Different types of yoghurt were produced from CaM and CaM:EM mixtures (i.e., pure CaM, 1.5CaM:1EM, and 1CaM:1.5EM) with starter s1 and s2, respectively. During the study, the panellists identified no significant difference between colours of the different samples as reported by Stahl et al. [107]. However, mixing EM with CaM (1.5CaM:1EM, 1CaM:1.5EM, *v*/*v*) improved the sensory profile (flavour, texture, taste and overall acceptability) of CaM yoghurt and increased both the marketability of CaM and EM.

## 5. Conclusions and Future Trends

During the past decades, undeniable efforts in the scientific community have been made to evaluate the effect of mixing different species of milk to produce yoghurt in order to evaluate quality features of the resulting products. Our literature review revealed that it has been fully demonstrated by different authors that the knowledge of how (i) the animal species from which the milk originates and (ii) the proportions of each species of milk used in the mixture influence the quality of yoghurt. Similarly, proportions necessary to design targeted products with improved physicochemical, nutritional, functional and sensory qualities are also discussed and corroborated with those highlighted in our previous review on cheese [108].

Nonetheless, further research on milk mixtures to produce yoghurt is required to obtain a clear overview of potentialities in nutritional, functional and sensory properties, among other characteristics of the resulting products. For example, little research has been done on:

- The understanding of the impact of the association of milk from different species on molecular structure and interactions of components (e.g., fat, proteins) during manufacture. This is essential for the quality attributes of yoghurts because texture, physicochemical properties, flavour, colour, nutritional profile, and bioavailability of nutrients, among other characteristics, are highly dependent on the microstructure.
- Nutritional effects in human diet. These yoghurts could potentially open new avenues by modifying the microbiome composition and altering the function of the host, due to the potentiality of incorporating LAB with specific probiotic effects.
- The shelf life of yoghurt, as these products generally present short life when compared to other fermented products (e.g., cheese).

We expect that this review will contribute to increase the interest of the scientific and industrial communities in developing and improving the quality of yoghurts processed from blending milks of different mammalian species. The increase in research studies will enhance knowledge of these products and highlight possible specific health and technological benefits not yet identified in classical yoghurt that will benefit both consumers and the industry, respectively, in both well-being and profits.

## Figures and Tables

**Table 1:** Approximate composition of milk from the various species.

	Camel Milk	Cow Milk	Goat Milk	Ewe Milk	Buffalo Milk	References
pH	6.38–6.65	6.50–6.70	6.55–6.69	6.51–6.85	6.61–6.81	[10,25,27,28,29,30,31]
Moisture (g/100 g)	86.6–90.4	87.00–88.1	83.67–88.3	81.5–83.3	82.3–84.0	[10,27,28,30,32]
Protein (g/100 g)	2.95–3.25	3.23–3.50	2.9–3.83	6.21–6.30	2.7–4.6	[10,27,28,30,33,34]
Fat (g/100 g)	2.65–3.60	3.60–3.67	3.8–5.30	7.62–7.90	5.3–9.0	[10,27,28,30,33,34]
Ash (g/100 g)	0.79–0.83	0.65–0.70	0.73–0.88	0.90–0.98	0.7–0.8	[10,27,28,30,33,35,36]
Lactose (g/100 g)	4.05–4.40	4.78–4.90	4.08–4.73	3.7–4.90	3.2–4.9	[10,27,28,30,33,34]
Acidity (g/100 g)	0.13	0.16–0.19	0.14–0.18	0.22–0.25	–	[10,27,28,37]
Casein (g/100 g)	2.10–2.30	2.28–3.27	2.14–3.18	3.78–5.20	3.02–3.2	[30,38,39,40]
Ca ^a^	106–120	112–120	126–198	195–200	147–220	[10,30,35,41]
Mg ^a^	12–14	7–11	13–36	18–21	2–16	[10,30,35,41]
P ^a^	63–90	59–92	97–153	124–158	102–293	[10,30,35,41]
Na ^a^	69–73	45–58	38–58	44–58	47	[10,30,35,41]
K ^a^	156–173	106–150	190–242	136–140	112	[10,30,35,41]
Fe ^a^	0.17–0.26	0.07–0.46	0.55	0.72–1.22	0.17	[10,30,41]
Cu ^a^	0.12–0.17	0.08–0.22	0.30	0.40–0.68	0.02	[10,30,41]
Zn ^a^	0.44–0.6	0.3–3.8	0.43–3.4	5.2–7.47	0.5	[10,30,41]
Mn ^a^	0.02–0.09	0.02–0.06	0.03–0.08	0.05–0.09	–	[10,15,41]
Vitamin A ^b^	0.01–0.05	0.06–0.37	0.04–0.54	0.08–0.64	0.07	[10,28,30,32,38,42]
Vitamin C ^b^	2.3–18.4	0.02–0.94	1.29–2.00	4.16–4.30	2.5	[10,15,30,34]
Vitamin D ^c^	–	0.08	0.06	0.18	–	[28,42]
Vitamin E ^b^	0.03	0.08–0.11	0.04	0.11–0.12	0.19	[28,30,32,42]
Vitamin B1 ^b^	0.03–0.60	0.04–0.05	0.05	0.07–0.08	0.05	[10,28,30,32,38,42]
Vitamin B2 ^b^	0.04–0.80	0.17–0.20	0.14–0.17	0.30–0.35	0.11	[10,28,30,32,38,42]
Vitamin B3 ^b^	0.46	0.09–0.13	0.20–0.23	0.41–0.42	0.17	[10,28,30,32,42]
Vitamin B5 ^b^	0.08	0.34–0.43	0.31	0.41–0.46	0.15	[10,28,30,32,42]
Vitamin B6 ^b^	0.05	0.04–0.05	0.04–0.05	0.06–0.08	0.33	[10,28,30,32,42]
Vitamin B8 ^c^	–	2.00–2.5	1.75–2.00	2.5	13	[28,30,32]
Vitamin B9 ^c^	4.10	5.30–8.5	1.00	5.00–6.00	0.6	[10,28,30,32,42]
Vitamin B12 ^c^	1.50–2	0.5–1.35	0.06–1.36	0.66–5.71	0.4	[10,28,30,32,38,42]
C4:0 ^d^	0.66–1.0	3.3–3.9	1.97–2.6	3.07–4.0	2.28–2.52	[10,15,31,34]
C6:0 ^d^	0.37	1.6–2.5	2.03–2.9	2.6–3.44	1.82–2.04	[10,15,31,34]
C8:0 ^d^	0.23–0.5	1.3–15	2.28–3.04	2.5–3.27	1.29–1.57	[10,15,31,34]
C10:0 ^d^	0.1–0.90	3.0–3.2	8.4–11.0	5.54–9.73	2.74–3.56	[10,15,31,34]
C12:0 ^d^	0.5–0.79	3.1–3.6	3.3–6.18	3.7–4.92	2.91–3.55	[10,15,31,34]
C14:0 ^d^	10.0–12.5	9.5–11.1	7.71–11.2	9.85–11.9	6.11–7.49	[10,15,31,34]
C16:0 ^d^	26.6–31.5	26.5–27.9	23.2–34.8	22.5–28.2	27.5–36.3	[10,15,31,34]
C16:1 ^d^	9.0–10.4	1.5–2.3	1.0–2.7	0.74–2.2	0.35–1.69	[10,15,31,34]
C18:0 ^d^	12.2–14.0	12.2–14.6	5.77–13.2	8.51–12.6	10.02–12.28	[10,15,31,34]
C18:1 ^d^	19.1–26.3	21.1–29.8	15.4–28.5	17.8–23.0	22.93–25.39	[10,15,31,34]
C18:2 ^d^	2.94–3.4	1.4–2.5	2.2–4.34	2.1–3.57	1.56–1.86	[10,15,31,34]
Ala ^e^	2.7–2.8	3.41–3.5	3–3.39	2.4	2.89–2.91	[10,36,43,44,45,46,47,48]
Arg ^e^	3.8–3.9	3.7–4.06	3.90	–	2.18–2.51	[10,36,43,44]
Asp ^e^	7.6–6.4	7.6–7.9	7.8–7.19	6.5	7.04–7.09	[10,36,43,44,46,47,48]
Glu ^e^	19.5–23.9	19.66–21.8	5.23–23.2	14.5	19.36–19.64	[10,36,43,44,46,47,48]
Gly ^e^	1.3–1.7	1.75–2.1	1.75–1.8	3.5	1.68–1.7	[10,36,43,44,46,47,48]
His ^e^	2.5–2.7	2.8–3.30	3–3.53	6.7	2.17–2.26	[10,36,43,44,46,47,48]
Ileu ^e^	5.0–5.4	4.54–6.4	4.2–4.61	4.6	4.68–5.56	[10,36,43,44,46,47,48]
Leu ^e^	9.5–10.4	9.44–10.4	8.7–9.80	9.7–9.9	8.74–8.97	[10,36,43,44,46,47,48]
Met ^e^	2.5–3.6	2.48–2.7	1.8–2.24	2.7	2.16–2.45	[10,36,43,44,46,47,48]
Phe ^e^	4.6–5.6	4.73–5.2	4.8–5.04	4.2–4.3	4.05–5.16	[10,36,43,44,46,47,48]
Pro ^e^	11.1–13	8.99–10.0	8.93–9.6	16.2	9.21–9.32	[10,36,43,44,46,47,48]
Ser ^e^	4.2–5.8	5.24–5.6	4.39–4.8	3.4	4.81–5.56	[10,36,43,44,46,47,48]
Thr ^e^	4.3–5.2	4.11–5.1	3.98–4.5	4.2–4.4	3.95–4.12	[10,36,43,44,46,47,48]
Tyr ^e^	4.0–4.5	5.3–5.67	4.5–4.67	3.7–3.8	3.53–3.9	[10,36,43,44,46,47,48]
Val ^e^	6.1–6.9	5.24–6.8	4.8–6.04	6.2–6.4	5.23–5.42	[10,36,43,44,45,46,47,48]

^a^ Mineral contents (mg/100 g of milk); ^b^ Vitamin contents (mg/100 g of milk); ^c^ Vitamin contents (µg/100 g of milk); ^d^ Fatty acid composition (g/100 g of total fatty acids); ^e^ Amino acid composition (g/100 g of total protein).

**Table 2:** A summary overview of some studies conducted on yoghurts containing mixtures of milk coming from different animal species.

Product	Country	Milk Mixture	Milk Ratios Studied	Breed	Coagulation	Ripening/Storage	Weight	Cheese Shape/Mould Shape	Reference
Stirred yoghurt	Spain	CM and GM	Pure CM, pure GM, 3CM:1GM, 1CM:1GM and 1CM:3GM (*v*/*v*)	Caprine (Murciano–Granadina) Bovine (Friesian)	Starter culture (MY900, RhodiaFood, Dangé Saint Romain, France), *containing Streptococcus thermophilus* and *Lactobacillus delbrueckii* ssp. bulgaricus (concentration of 0.08 U/L)	28 days at 4 °C	-	-	Vargas et al. [60]
	Turkey	CM and GM	1CM:1GM (*v*/*v*)	-	0.1 g/L of frozen pellets (starter culture DI-PROX TY 973, Bioprox, France)	15 days at 4 °C	200 mL	Cups	Kucukcetin et al. [61]
Set type yoghurt	Turkey	CM and GM	Pure GM, 1CM:1GM and 1CM:2.33GM (*v*/*v*)	-	The culture consisted of *Lb. acidophilus* (5.8 × 10^8^ cfu/g), *Str. thermophilus* (3.8 × 10^8^ cfu/g) and *B. bifidum* (7.2 × 10^7^ cfu/g)	14 days at ± 4 °C	-	-	Uysal et al. [62]
	Turkey	GM and EM	1CM:1GM (*v*/*v*)	Goat (Shami) Ewe (Awassi)	CH-1 and YF-3331 type cultures	21 days at 4 °C	-	Polystyrene plastic cups	Güler and Gürsoy-Balci [63]
	Sudan	CaM and EM	Pure CaM, 2.33CaM:1EM and 1CaM:2.33EM (*v*/*v*)	-	YC-X11 Thermophilic Yoghurt and CH-1 Thermophilic Yoghurt Cultures	29 days at 4 °C	-	-	Ibrahem and El Zubeir [64]
	Greece	GM and EM	Pure GM (Alpine), pure GM (Thiva), pure EM (Lacaune), 1GM(ALacaune):1EM(Alpine) and 2.33GM(Thiva):1EM(Lacaune)	Ovine (Lacaune) Caprine (Thiva and Alpine)	Starter cultures of *Streptococcus thermophilus* and *Lactobacillus bulgaricus*	1 day at 5 °C	250 mL	Cups	Kaminarides and Anifantakis [65]
	Brazil	CM and EM	Pure CM, pure EM, 3CM:1EM, 1CM:1EM and 1CM:3EM	-	Starter (Yo-Flex, Chr Hansen, Valin-hos, SP, Brazil) and *Lb. acidophilus* cultures were inoculated at concentration of 1% (*v*/*v*) and 5% (*v*/*v*), respectively	28 days at 4 °C	200 mL	Sterile flask	Vianna et al. [66]
	Pakistan	GM and EM	Pure GM, pure EM, 3GM:1EM, 1GM:1EM and 1GM:3EM	-	Starter culture CSK Y104	28 days at 4 °C	-	Polypropylene cups	Bano et al. [67]
Bioghurt	Turkey	CM and GM	Pure GM, 1CM:1GM and 1CM:2.33GM (*v*/*v*)	-	Streptococcus salivarius ssp. thermophilus and Lb. acidophilus starter cultures	1, 7, and 14 days at 4 °C	-	-	Kavas et al. [68]
Concentrated yoghurt (lebneh)	Lebanese	CM and GM	Pure CM, pure GM, 1CM:1GM, 1.5CM:1GM, 1.33CM:1GM, 4CM:4GM and 9CM:1GM	Cow (local breed); Goat (local breed)	2% starter culture (*Streptococcus thermophilus* and *Lactobacillus delbrueckii* subsp. bulgaricus in equal proportions, Yo-Mix 505 LYO 200 DCU lyophilized powder)	4 °C	500 g	Disinfected labelled PVC containers (500 g), closed with an aluminium foil	Serhan et al. [69]

**Table 3:** Examples of principal conclusions of studies concerning the effect of mixing milk from different species at different proportions on physicochemical and biochemical properties of yoghurts.

Product	Principal Conclusion	Reference
Set type yoghurt	EM and GM mixtures showed no significant differences in acidity and pH. The addition of EM into GM increased the protein content to 4.1%. Mixing EM and GM improved the composition, serum separation and pH of the resultant yoghurts.	Kaminarides and Anifantakis [65]
	The addition of GM in EM did not affect acidity and pH of yoghurts. EM addition into GM increased protein, fat, lactose, ash and total solids contents of yoghurt.	Bano et al. [67]
	Pure EM and 1CM:3EM yoghurts had greater values for total solids, protein, and lipid contents when compared to pure CM and 3CM:1EM yoghurts.	Vianna et al. [66]
	Increasing the percentage of EM into CaM exhibited: an increase in total solid, protein and fat content of yoghurts no differences between yoghurts.	Ibrahem and El Zubeir [64]
	Mixing GM with CM did not affect the fat and total solids contents of the samples. The kind of milk had a little effect on yoghurt acidity except samples (2.33GM:1CM and 1GM:1CM, *v*/*v*) made by skim milk powder addition.	Uysal et al. [62]
	Mixing EM with GM had an effect on cholesterol contents.	Bernacka et al. [70]
Concentrated yoghurt (Labneh)	Labneh samples made with 20, 30, 40 and 50% of GM presented intermediate moisture, protein, fat and ash contents. The replacement of CM by GM tended to reduce short fatty and linoleic acids and to increase palmitoleic acid.	Serhan et al. [69]
Stirred yoghurt	Adding GM to CM tended to decrease pH. Moreover, yoghurts containing 50% or less GM showed a slowest pH decrease.	Vargas et al. [60]
	The rate of the acid development in pure GM yoghurt was faster than that noticed within pure CM and 1GM:1CM samples. The addition of GM into CM resulted in yoghurt with decreased viscosity. The water holding capacity obtained from GM was lower than that obtained from CM and 1GM:1CM.	Kucukcetin et al. [61]

**Table 4:** Examples of principal conclusions of studies concerning the effect of mixing milk from different species at different proportions on microbiological properties of yoghurts.

Products	Principal Conclusion	Reference
Set Type Yoghurt	Adding GM into EM had a significant effect on *delbrueckii* spp. bulgaricus, *Streptococcus thermophilus* and LA-5.	Vianna et al. [66]
	*Streptococcus thermophilus* and *Lactobacillus delbrueckii* subsp. bulgaricus activities in yoghurt made with CM and the mixture CM:GM were slower than that stated within GM.	Kucukcetin et al. [61]
	Mixing GM with CM had no significant effect on the viable counts of *Lb. acidophilus*, *Str. thermophilus*, *B. bifidum* and yeasts–moulds.	Uysal et al. [62]

**Table 5:** Bacterial population detected in the different yogurt samples.

Yogurt Samples	Bacterial Population Exhibited (cfu/g)
pure CM	8.2, 12.2 and 8.6
3CM:1EM	7.0, 12.6 and 9.0
1CM:1EM	9.2, 12.5 and 8.3
1CM:3EM	7.0, 13.2 and 9.5
pure EM	7.0, 10.5 and 9.0

**Table 6:** Examples of principal conclusions of studies concerning the effect of mixing milk from different species at different proportions on rheological and sensory properties of yoghurts.

Products	Principal Conclusion	Reference
Set type yoghurt	Yoghurts, which contained 100, 50 and 30% of EM, showed higher levels in term of flavour when compared to pure GM. The addition of EM into GM improved the textural characteristics of yoghurts.	Kaminarides and Anifantakis [65]
	Mixing EM to GM up to 25% level improved the colour of yoghurt. Increasing EM into GM resulted in decreasing flavour score. Increasing EM content provided a better texture (higher score for acceptability)	Bano et al. [67]
	1EM:1CM sample was the only treatment that exhibited the ideal consistency for consumers suggesting. Sensory properties were positively influenced by the higher EM ratio in yoghurt samples (3EM:1CM).	Vianna et al. [66]
	The addition of EM to CaM improves the quality and acceptability of camel milk yoghurt.	Ibrahem and El Zubeir [64]
	Mixing GM with CM did not affect the texture of yoghurts. Milk type affected whey loss values from the coagula of all the yoghurts except samples (pure GM and 2.33GM:1CM) made by skim milk powder addition.	Uysal et al. [62]
	EM:GM, EM:CM and CM:GM (*v*/*v*) yoghurts had significant differences regarding taste, smell, colour and consistency. Yoghurts formulated with pure GM, mixed CM:EM pure CM was characterized by the highest stability of curd.	Bernacka et al. [70]
Concentrated yoghurt (Labneh)	Samples with 1GM:1.5CM (*v*/*v*) mixtures were the most preferred by the sensory panel.	Serhan et al. [69]
Stirred yoghurt	The addition of GM in yoghurt increased whiteness index and decreased gel firmness, consistence and syneresis. Pure GM and 1GM:1CM (*v*/*v*) yoghurts had a smaller number of junction points and larger pores. The milk type influenced the mean perimeter and the number of grains. Mixing GM with CM yoghurt improved the sensory qualities of samples.	Vargas et al. [60]
